# Supplementary figures and images for: Negshell casting: 3D-printed structured and sacrificial cores for soft robot fabrication
Source: PLoS One. 2020 Jun 12;15(6):e0234354. doi: 10.1371/journal.pone.0234354 (PMC7292399; doi:10.1371/journal.pone.0234354)

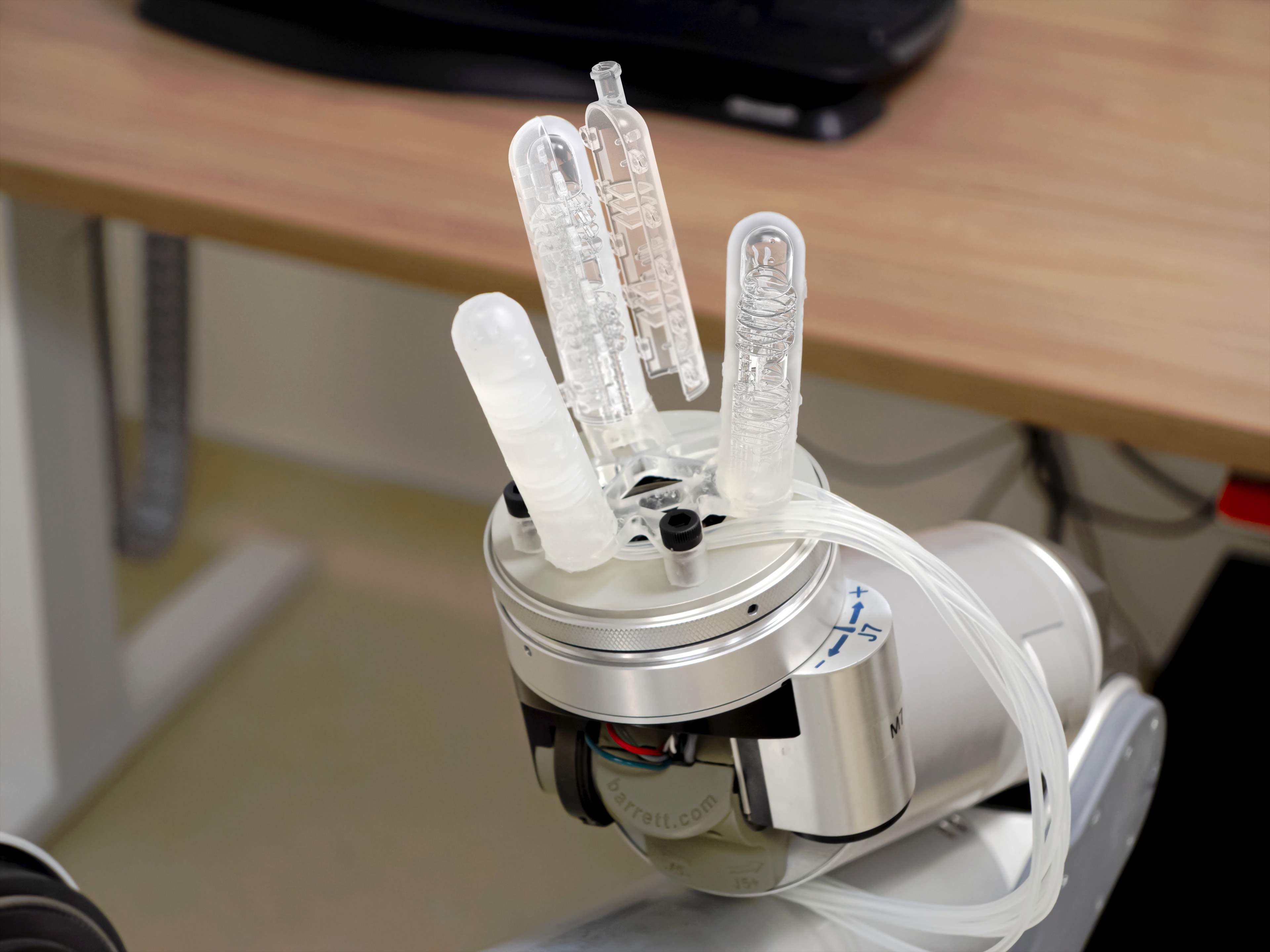

Supplement: S1 Fig — (JPG) [file pone.0234354.s005.jpg]
