# Supplementary material for: Negshell casting: 3D-printed structured and sacrificial cores for soft robot fabrication
Source: PLoS One. 2020 Jun 12;15(6):e0234354. doi: 10.1371/journal.pone.0234354 (PMC7292399; doi:10.1371/journal.pone.0234354)
Supplement: Attachment — Submitted filename: Negshell PLOS One Rebuttal Letter.pdf [file pone.0234354.s006.pdf]

Dear Dr.Ning Cai & Reviewers,

First of all, we would like to thank the editors and all reviewers for their valuable input for our paper. We present here a revised copy of our manuscript. We would like to point out issues raised in the original review and letter with the following:

1. **To enhance the reproducibility of your results, we recommend that if applicable you deposit your laboratory protocols in protocols.io**
   Response: We have opted to use GitHub as our repository for the project instead as protocols.io requires a paid plan for projects larger than 100MB and has a strict layout, which is not ideal for engineering projects. We host our data and protocols on GitHub Pages instead: https://negshell.github.io/, this gives us much more flexibility in terms of presentation and content.
2. **Please ensure that your manuscript meets PLOS ONE's style requirements**
   Response: We have read through our manuscript carefully to ensure that it complies to the PLOS One formatting guidelines and have touched up any mistakes found.
3. **We note that Figures in your submission contain copyrighted images**
   Response: We were unsure which figure had copyrighted images, as all figures were generated by ourselves. Figure 17 is the only image that has possibly copyrighted material: the logo of a whiteboard marker company. We have edited the Figure to not contain the logo. Any other logo (Rombolabs, VA, CLiMB, etc. are our own and we have the right to use them along with our affiliation with the respective groups)
4. **From Reviewer 1:**
   1. The fragment of the negshell may do harm to the soft material, and reduce the life of the robot.
   2. Also, the fragment of the negshell may block the inner channel of the robot.
   3. In my opinoin, the authors should consider the circle life of the actuator due to the questions I mentioned above.
   Response: Reviewer 1 has brought up a good point about the longevity of negshell-produced soft robots. We agree that the fragments can cause issues if cross-hatch is designed improperly. Blockage can be mitigated by making the pattern smaller than the smallest orifice in the design thus even if a fragment is lodged in an orifice, the fragment will have perforations to allow fluids to travel through. As for the sharpness of the fragments, we argue that as the fragments are created while physically crushing the negshell cores during the fabrication process, the soft material used must be able to mechanically withstand them anyways. Our fabrication process would be invalidated if they were in fact causing damage to the structure, which our prototypes and experiments show otherwise. Furthermore, we have found that the 0.4 mm thickness negshell cores are in fact soft and compliant themselves, though this is not covered in the paper as we did not rigorously characterize them. We have addressed these issues in our revised manuscript in the Discussion section.

We hope that the revisions satisfy the reviewers concerns, and are welcome to any additional comments.

Thank you & Best regards,
Pornthep Preechayasomboon
Eric Rombokas
